# Associations between intimate partner violence and women’s labor market outcomes in Nigeria

**DOI:** 10.1186/s41256-024-00362-1

**Published:** 2024-06-20

**Authors:** Derek S. Brown, Samantha McNelly, Melissa Meinhart, Ibrahim Sesay, Catherine Poulton, Lindsay Stark

**Affiliations:** 1 George Warren Brown School, Washington University in St. Louis, Goldfarb Hall, Room 241, Campus Box 1196, One Brookings Drive, St. Louis, MO 63130 USA; 2 UNICEF, New York, USA

**Keywords:** Costing, Violence, Intimate partner violence, Humanitarian, Economics

## Abstract

**Background:**

Little is known regarding economic impacts of intimate partner violence (IPV) in humanitarian settings, especially the labor market burden. Examining costs of IPV beyond the health burden may provide new information to help with resource allocation for addressing IPV, including within conflict zones. This paper measures the incidence and prevalence of different types of IPV, the potential relationship between IPV and labor market activity, and estimating the cost of these IPV-associated labor market differentials.

**Methods:**

The association between labor market outcomes, IPV experience, and conflict exposure among women ages 15–49 in Nigeria were studied using the 2018 Nigeria Demographic and Health Survey and 2013–17 Uppsala Conflict Data Program data. Descriptive analysis was used to identify patterns of IPV and labor outcomes by region. Based on this, multivariable logistic regression models were used to estimate the association between labor market participation and lifetime IPV exposure. These models were combined with earnings data from the United Nations Human Development Report 2021/2022 and a top-down costing approach to quantify the impacts in terms of lost productivity to the Nigerian economy.

**Results:**

Substantial differences in IPV exposure and labor market outcomes were found between conflict and non-conflict-affected areas. Women with past year or lifetime exposure to physical, emotional, or “any” IPV were more likely to withdraw from the labor market in the past year, although no differences were found for sexual IPV or conflict-affected regions. We estimate an average reduction of 4.14% in the likelihood of working, resulting in nearly $3.0 billion USD of lost productivity, about 1% of Nigeria’s total economic output.

**Conclusions:**

Increased odds of labor market withdraw were associated with several measures of IPV. Withdrawal from the formal labor market sector has a substantial associated economic cost for all of Nigerian society. If stronger prevention measures reduce the incidence of IPV against women in Nigeria, a substantial portion of lost economic costs likely could be reclaimed. These costs underscore the economic case, alongside the moral imperative, for stronger protections against IPV for girls and women in Nigeria.

## Introduction

The epidemic of gender-based violence (GBV) is exacerbated during humanitarian emergencies, with intimate partner violence (IPV) being the most prevalent form of GBV experienced by conflict-affected women and girls [1, 2]. Although IPV is recognized as a persistent problem with wide-ranging impacts in humanitarian settings [3], little is known regarding economic impacts of IPV in humanitarian settings [4]. Existing studies tend to focus on predictors of IPV [5], but quantifying the burden of IPV with reliable cost estimates may help policymakers prioritize resources to reduce the incidence of IPV.

‬Although a limited number of studies present health-related costs of IPV in humanitarian settings [6, 7], there are few estimates of labor-related costs. IPV may lead to absenteeism and lost productivity, which are borne by individuals, households, businesses, and society. These costs are particularly salient given that labor force participation is a key component of gross domestic product (GDP) and economic stability [8]. If there is a relationship between IPV and labor market engagement, research may elucidate the links between social policy, public health, and economic output. Some studies have considered whether women’s employment and labor force engagement is associated with the risk of experiencing IPV, indicating links between employment, education, social norms, and IPV victimization [9, 10]. Other research has considered whether IPV impacts employment and labor force engagement, although research is scant in lower and middle income countries (LMICs) and humanitarian settings [9, 11, 12].

While many factors influence labor force participation, experience of or exposure to violence, including IPV, are important determinants [13]. IPV may impact survivors’ work in several ways, including adverse health outcomes or injuries, partner control or monitoring, or partner control over materials or production inputs. Research has demonstrated that humanitarian-impacted women may be more likely to be employed, but in less skilled positions, and people living in areas with a large number of displaced persons receive lower wages and have fewer employment opportunities than those living in less affected areas [14].

Nigeria, Africa’s most populous country and largest economy, is currently experiencing several ongoing internal conflicts (namely the Boko Haram insurgency) and is home to 3.1 million displaced persons [15, 16]. Nigeria also has high rates of IPV [17], with a lifetime prevalence estimated to be 25% and over 70% in the state of Taraba [18]. The Nigerian Violence Against Persons Prohibition Act (VAPP) was passed in 2015, but to date, has been adopted by only 17 out of 37 states, highlighting a lack of uniformity of IPV protections and resources for survivors [19–21]. In 2020, all Nigerian state governors announced a GBV state of emergency, indicating the severity of the issue, but the implementation of policies to address the high prevalence of IPV still lags, and there is unequal adoption and enforcement of GBV-related laws [19].

Nigerian men and women are primarily involved in informal labor [22], with 36% engaged in agriculture [22], 29.7% of women and 42% of men. Seasonal work is common [23], and women experience greater seasonal shifts in employment than men, 14.4% and 12.7%, respectively, indicating more precarious employment and income for women [22]. Education is an important predictor of future employment for all, and of labor force participation for women [24]. However, women earn less than men for the same level of education, highlighting gender wage disparities [24]. Nigeria provides a unique case study for an analysis of the impacts of IPV on labor market outcomes due to its multiple ongoing conflicts, dynamic but gendered economy, high rates of IPV, and a vested government interest in reducing the prevalence of IPV.

In this paper, we seek to expand the narrow existing evidence base linking IPV exposure with labor force participation by measuring the incidence and prevalence of IPV, the potential relationship between IPV and labor market activity, and estimating the cost of these IPV-associated labor market differentials. Given that IPV risks, patterns, labor markets, and policy strategies all differ for humanitarian zones, analyses are conducted separately for conflict and non-conflict affected populations of women ages 15–49 in Nigeria.

## Methods

### Study design and setting

The setting for this study was 2018 Nigeria, with results about IPV, labor market analysis, and economic costs pertaining to women. Our analysis was based in a combination of public health investigation of IPV in humanitarian settings [25] and basic labor economic theory [26]. We hypothesized that experiencing IPV added an additional negative cost to women who were engaged in the workforce. The mechanism for these costs was myriad and not well captured by the questions in the dataset but could include several possible pathways. For example, a violent partner or spouse may restrict a woman’s activities and bar them from working. Alternatively, IPV could reduce a woman’s ability to perform certain tasks and limit their employment in the formal sector. Moreover, IPV may result in reduced ability to concentrate, lower productivity, increased absenteeism, or generally decreased labor performance. In other words, IPV is likely to have a negative effect on a woman’s productivity, making them less likely to be able to obtain a job, or to only find worse types of employment, which are not desirable.

To examine this hypothesis, we conducted a series of descriptive analyses around the prevalence and incidence of IPV, types of IPV, and its linkage to employment. Whether or not the associations between IPV and employment were further impacted by residing in a conflict-affected area was previously unknown; however, given the deleterious impacts of IPV in humanitarian settings [1, 2], and the economic strain on humanitarian populations generally [14], we further assessed if the labor impacts were different by conflict and non-conflict affected regions. For policy context, we also estimated the associated economic costs of labor market impacts using a human capital measure (Becker, 1965) of lost earnings.

### Data collection

The main data source for this study was the nationally representative domestic violence module of the 2018 Nigeria Demographic and Health Survey (NDHS) for women ages 15–49. For safety purposes, only one woman per eligible household was surveyed and women must have been married at some point in their life in order to be eligible. NDHS uses an established series of questions for all respondents randomly selected for the domestic violence module, giving women additional opportunities to disclose experiences of violence.

Two secondary sources of data were used for this analysis to determine the conflict exposure variable and to establish earnings estimates. Conflict exposure of NDHS female respondents was determined based on integrating Uppsala Conflict Data Program (UCDP) GIS data to determine whether NDHS enumeration areas were within 50 km of a conflict event in the five years prior to NDHS data collection, via integrating the GIS coordinates of conflict related events between 2013–17 using UCDP data. The final source of data was the Gender Development Index in the United Nations Human Development Report 2021/2022 which was used to identify earnings data by gender in Nigeria.

### Exposure variables

Lifetime and past-year emotional, physical, and sexual IPV were measured. A respondent was considered as having experienced *physical IPV* if an affirmative response was given to ever having been pushed, shaken, slapped, punched, kicked, dragged, strangled or burned (attempted or actual), threatened with a knife, gun, or other weapon, or had her arm twisted or hair pulled by a past or current husband or partner. A respondent was considered as having experienced *sexual IPV* if she responded affirmatively to having been physically forced to have sex or engage in other unwanted sexual acts by a current or former husband or partner. *Emotional IPV* was said to have occurred if the respondent indicated ever having been humiliated, threatened, insulted, or made to feel bad by her current husband or partner. (Respondents were not asked about emotional violence perpetrated against them by any past partners.) Based on respondent indications of the temporality of the abuse experienced, each type of violence was used to create two variables: past-year and lifetime experiences of sexual, physical, or emotional violence. Finally, all types of violence were collapsed into two composite measures of IPV: any lifetime IPV (sexual, physical, or emotional), or any past-year IPV (sexual, physical, or emotional). Approximately 10% of the sample had missing data for the IPV questions, but no meaningful differences were found between the characteristics of respondents with missing and non-missing IPV data.

### Outcome variable

Labor market participation was measured by whether the respondent had worked in the last year, the seasonality of their work, and how they were compensated. Respondents provided binary, yes/no responses, to whether they were currently working or had worked in the past 12 months. Using questions asked in the NDHS about seasonality of work (respondents who had worked in the last 12 months could specify having worked year-round, seasonally, or occasionally) and the type of earnings from work (unpaid work, cash only, in-kind only, or both cash and in-kind), we constructed our primary dependent variable to reflect whether or not a respondent had worked year round in the past 12 months for cash. Respondents also indicated the type of work in which they typically engaged according to standard NDHS categories: professional/technical/managerial, clerical, sales, services, skilled or unskilled manual labor, agriculture, or other. (Data on type of work, seasonality, and type of earnings were available only for respondents who reported “yes” to currently working.) Finally, respondents indicated their highest level of education as none (no education), primary, secondary, or higher education.

### Control variables

Table 1 provides an overview of the individual, partner, and household-level covariates used for the multivariable analysis. Individual-level covariates included age (continuous), marital status (binary), education level (categorical), number of children (continuous), number of children under 5 (continuous), and Christian religion (binary); at the household level, covariates included household size (continuous), female head of household (binary), and urban residence (binary) [27].

**Table 1:** Study variables

*Core variables*		*Covariates*	
*Conflict exposure*	Located in an enumeration area that was within 50 km of a conflict event between 2014–2018	*Individual characteristics*	Age; currently married (yes or no); number of children born; number of children under 5; Christian religion (yes or no); level of education (no education, primary, secondary, or higher)
*Physical IPV*	Experience was measured if a respondent reported her partner pushed, shook or threw something at the respondent; slapped the respondent; punched with fist or hit the respondent with something harmful; kicked or dragged the respondent; strangled or burnt the respondent, twisted respondent's arm or pulled her hair; and/or; threatened the respondent with a knife, gun, or other weapon	*Household characteristics*	Household size; female headed household (yes or no); urban (yes or no)
*Emotional IPV*	Experience was measured if a respondent reported her partner humiliated the respondent; threatened the respondent with harm, and/or; insulted or made the respondent feel bad		
*Sexual IPV*	Experience was measured if a respondent reported her partner physically forced respondent into unwanted sex; and/or forced respondent to perform other unwanted sexual acts		
*Any IPV*	Experience was measured based on if a respondent reporting experiencing physical, emotional, and/or sexual intimate partner violence		
*Working*	Past year (12 months) work experience in year-round and cash-based employment		

All IPV exposure variables were measured as lifetime (ever experienced) and also if experienced in the past year (12 months). Past-year variables reflect IPV experienced by the respondent's current partner only. Lifetime emotional violence was asked only for the current husband/partner, not any past husband/partners. Physical and sexual violence include all lifetime partners

### Data processing and analysis

We used a series of steps to estimate the total labor market costs of IPV among conflict and non-conflict affected women in Nigeria ages 15–49. First, using the NDHS data, we estimated the prevalence of IPV experiences, conflict exposure, and work experience of Nigerian women. Descriptive statistics were examined for the full national sample, and separately for conflict-affected and non-conflict affected subgroups. Tests of statistical differences between conflict and non-conflict affected groups were conducted using chi-square and proportions tests. Next, we examined the direct correlation between IPV experience and conflict exposure, focusing on women who had worked in year-round and cash-based employment during the past 12 months, again testing for differences using chi-square tests. Third, using multivariable logistic regression models, we estimated the odds ratios (ORs) of negative labor market impacts (not being engaged in year-round and cash-based employment during the past 12 months) based on IPV experience and conflict exposure. Models were run separately for lifetime and past-year IPV, four definitions of IPV (sexual, physical, emotional, and any), and for the full sample, and by conflict and non-conflict affected subsamples of women. Tests of significant differences between conflict and non-conflict affected regions were conducted using interaction effects with each IPV measure. For costing, ORs were converted to estimated probabilities of not working, reflecting the marginal damage of IPV in the labor market. All analyses of NDHS data were conducted in Stata 17.0 [28], using complex survey models which accounted for the NDHS design.

### Economic cost model and analysis

The economic costs of labor market impacts may be estimated using a variety of different approaches and economic models. Given the available measures in the data, we followed similar studies [5, 6] and estimated lost productivity as the value of forgone earnings in the formal sector (Becker, 1965), a basic human capital model of earnings. This model estimates the impacts by paid earnings and does not reflect any substitution effects into the informal sector, such as possible increased “home productivity” which may result from reduced formal employment. The human capital model posits that the economic value of an individual in the workplace is approximated by median earnings for individuals. Specifically, if one person with a given set of characteristics drops out of the labor force, the lost societal earnings would be equal to median earnings for a comparable individual. Adjustments for seeking work but not being employed are not necessary, as a localized median wage already reflects general labor market condition, including factors such as unemployment, overall economic activity and development, and cultural norms.

Costs were estimated in Microsoft Excel in several steps. First, the estimated prevalence of lifetime IPV was combined with aggregate population counts of Nigerian women in 2021, by age group, from the World Bank [29]. This generated estimates of the total number of Nigerian women ages 15–49 who were affected by IPV during their lifetime, for all of Nigeria, and by conflict and non-conflict affected regions. Because work and IPV patterns varied significantly by age, we retained the age categories [25] in reporting of economic costs. The estimated number of IPV victims was multiplied by the probability of not working from the logistic regression models to estimate the number of women who were no longer active in the labor force in the past year due to IPV exposure. Finally, the economic value of lost income was quantified by multiplying our estimates by median gender-specific earnings data ($3759 for women, $5800 for males) from the Gender Development Index in the United Nations Human Development Report 2021/2022 [30]. The latter reports 2021 female earnings in USD, based on 2017 purchasing power parity (PPP) exchange rates. Costing was conducted only for lifetime exposure to any IPV to avoid potential double counting, because subtypes of IPV are not mutually exclusive, nor are lifetime and past year IPV.

## Results

### IPV Experience by conflict-exposure history

Table 2 presents descriptive statistics on conflict exposure, experiences of IPV by type and temporality, level of education, and work experience. Nearly one-fifth of the population (19.76%) were determined to live in a conflict-affected area. IPV was widespread, with 38.37% of women indicating having experienced some type of IPV throughout their lives.

**Table 2:** IPV experience, conflict exposure and work experience among women ages 15–49 in Nigeria

		Full Sample (*N* = 10,678)	Conflict-affected subgroup (*n* = 2110)	Non-conflict affected sub-group (*n* = 8568)	Significance between conflict exposure
		%	%	%	y/n
*Intimate partner violence (n* = *8910, n* = *1781, & n* = *7129)*					
Lifetime IPV (any)		38%	39%	38%	
Lifetime IPV (emotional)		33%	35%	32%	*
Lifetime IPV (physical)		22%	21%	22%	
Lifetime IPV (sexual)		8%	11%	7%	***
Past year IPV (any)		31%	33%	30%	*
Past year IPV (emotional)		28%	31%	27%	**
Past year IPV (physical)		13%	14%	13%	
Past year IPV (sexual)		5%	7%	4%	**
*Education*					
Highest level of education	No education	32%	43%	30%	***
	Primary education	16%	14%	17%	
	Secondary education	41%	32%	43%	
	Higher education	11%	11%	11%	
*Work*					
Worked in year-round and cash-based employment during the past 12 months		41%	35%	42%	***
Currently working		68%	59%	70%	***
Past 12-month work		50%	62%	74%	***
Type of work (*n* = 7625, *n* = 1317, & *n* = 6308)	Professional, technical, managerial	9%	10%	9%	***
	Clerical	2%	3%	1%	
	Sales	49%	53%	49%	
	Services	10%	9%	10%	
	Skilled Manual Labor	6%	8%	5%	
	Unskilled manual labor	9%	2%	0%	
	Agriculture	24%	17%	26%	
	Other	0%	0%	0%	
Seasonality of work (*n* = 7625, *n* = 1317, & *n* = 6308)	Year round	76%	70%	77%	***
	seasonal	19%	23%	18%	
	occasional	6%	8%	5%	
Year-round work in the past year		54%	43%	57%	***
Type of earnings from work (*n* = 7625, *n* = 1317, & *n* = 6308)	Not paid	18%	13%	19%	***
	Cash only	69%	75%	68%	
	Cash and in-kind	12%	11%	12%	
	In-kind only	1%	1%	1%	

Note: *** *p* < 0.001, **p < 0.01, *p < 0.05. Cells related to significance that are blank indicate a *p*-value > 0.05. *IPV* intimate partner violence

The most common type of IPV reported both in the past year and over the course of the respondents’ lifetime was emotional, with 32.97% and 27.58% reporting lifetime and past-year experiences, respectively. The least common type of IPV reported was sexual, with 7.7% of women reporting lifetime sexual violence, and 4.84% reporting past-year sexual IPV.

Chi-square analyses identified statistically significant differences between conflict-affected and non-conflict affected groups in the experiences of most types of IPV. Conflict-affected women were more likely to experience many of the forms of IPV, including lifetime emotional IPV (64.68% vs. 32.39%, *p* < 0.05) and sexual IPV (11.01% vs. 6.86%, *p* < 0.001), as well as past year any IPV (33.41% vs. 29.98%, *p* < 0.01), emotional IPV (30.83% vs. 26.76%, *p* < 0.01), physical IPV (14.21% vs. 13.21%, *p *< 0.01), and sexual IPV (6.68% vs. 4.38%, *p* < 0.001).

### Work experiences by conflict-exposure history

Work experiences also differed significantly based on conflict-exposure. Nearly half (40.95%) of the total population worked year-round for cash during the past 12-months; however, among conflict-affected populations, this fell to 35.17%, while 42.38% of non-conflict affected persons were involved in such employment (*p* < 0.001). More than two-thirds (68.05%) of the total population were currently working at the time of the survey, whereas 58.86% of conflict-affected and 70.31% of non-conflict affected women reported currently working (*p* < 0.001). Most respondents indicated having worked in the past 12-months (71.41%), but this also varied significantly as 62.42% of conflict-affected respondents and 73.62% non-conflict affected women indicated having worked in the past 12-months (*p* < 0.001).

In the full sample, 54.13% of respondents indicated having worked year-round in the past year, while only 43.46% of conflict-affected respondents and 56.76% of non-conflict affected persons indicated working year-round in the past year (*p *< 0.001). Seasonality of work was significantly different for conflict and non-conflict exposed populations. Among all respondents, 75.8% indicated working year-round, 18.68% worked seasonally, and 5.52% worked occasionally. In the conflict-affected sub-group, 69.63% reported working year-round, 22.78% worked seasonally, and 7.59% worked occasionally, whereas in the non-conflict affected group, 77.09% worked year-round, 17.82% worked seasonally, and 5.09% worked occasionally (*p* < 0.001). Work earnings also differed significantly based on conflict exposure. In the full sample, 17.55% of respondents indicated not being paid for their work, 69.34% were paid in cash, 11.91% were paid in cash and in-kind, and 1.21% were paid only in-kind. In the conflict-affected sub-group, 12.91% reported not being paid, 74.87% were paid in cash, 11.24% were paid in cash and in-kind, and 0.99% were paid only in-kind, compared to the non-conflict affected sub-group where 18.52% of respondents were not paid, 68.18% were paid in cash, 12.05% were paid in cash and in-kind, and 1.25% were paid in-kind only (*p* < 0.001).

Finally, the type of work in which respondents engaged differed significantly based on conflict exposure, with greater percentages of conflict-affected persons engaging in professional/technical/ managerial, clerical, sales, and unskilled manual labor compared to non-conflict affected respondents. However, non-conflict affected respondents reported greater percentages of engagement in services, skilled labor, agriculture, and other forms of employment (*p* < 0.001).

### Labor force participation by IPV experience and conflict-exposure history

Table 3 presents estimates and chi-square analyses of differences in whether respondents had worked year-round for cash in the past 12-months and their IPV exposure status, for the full sample, and conflict-affected and non-conflict affected sub-groups. Across all respondents, significant differences were found between work experiences and all types and temporalities of IPV, except lifetime sexual violence. In the full sample, 43.78% of respondents who had experienced any lifetime IPV reported working year-round for cash in the past 12-months, compared to 49.02% of women who did not experience any lifetime IPV (*p* < 0.001). Nearly half of women who experienced lifetime emotional IPV (43.1%) reported working year-round in the past 12 months for cash, compared to 48.94% of women who did not experience any lifetime emotional IPV (*p* < 0.001). Similarly, 43.68% of respondents who disclosed lifetime physical IPV and 47.98% of women who did not, report working year-round for cash in the past 12-months (*p* < 0.05). When considering any past-year IPV, 41.69% of women who did experience any past-year IPV and 49.34% of women who did not report any past-year IPV worked year-round for cash in the past 12 months (*p* < 0.001). Of women who experienced past-year emotional IPV, 42.1% reported working year-round for cash in the past 12-months, compared to 48.91% of women who did not experience past-year emotional IPV (*p* < 0.001). Similarly, 40.37% of women who experienced past-year physical IPV and 48% of those who did not, report working year-round for cash in the past 12-months (*p* < 0.001). When considering past-year sexual IPV, 38.85% of women who reported, and 47.46% of women who did not report experiencing past-year sexual IPV indicated having worked year-round for cash in the past 12-months (*p* < 0.01).

**Table 3:** Percent of women ages 15–49 in Nigeria who have worked in year-round and cash-based employment during the past 12 months, based on IPV experience and conflict exposure among

	Full Sample (*n* = 8910)			Conflict-affected sub-group (*n* = 1781)			Non-conflict affected sub-group (*n* = 7129)		
	*No IPV*^+^	*IPV*^+^	*Significance between IPV experiences*	*No IPV*^+^	*IPV*^+^	*Significance between IPV experiences*	*No IPV*^+^	*IPV*^+^	*Significance between IPV experiences*
*Lifetime IPV*									
Any	49.02%	43.78%	***	42.10%	36.04%		50.58%	45.73%	**
Emotional	48.94%	43.10%	***	41.35%	36.86%		50.61%	44.81%	***
Physical	47.98%	43.68%	*	41.32%	33.45%	*p* = 0.0548	49.56%	46.01%	*p* = 0.0549
Sexual	47.49%	41.95%	*p* = 0.0575	41.07%	28.87%	*	48.93%	47.19%	
*Past year IPV*									
Any	49.34%	41.69%	***	41.77%	35.74%		51.00%	43.34%	***
Emotional	48.91%	42.10%	***	41.47%	36.04%		50.52%	43.88%	***
Physical	48.04%	40.37%	***	41.20%	30.54%	*	49.67uuuuuuu2%	42.99%	**
Sexual	47.46%	38.85%	**	40.58%	26.84%	*	49.05%	43.22%	

The corresponding form of IPV is described the respective row. *** *p* < 0.001, **p < 0.01, *p < 0.05. Cells related to significance that are blank indicate a *p*-value > 0.05. *IPV *intimate partner violence

Among the conflict-affected subgroup, there were significant differences between work experiences for people who reported lifetime sexual IPV, past-year physical IPV, and past-year sexual IPV. When considering lifetime sexual IPV, 28.87% of conflict-affected women who experienced this violence and 41.07% of conflict-affected women who did not, reported working year-round for cash in the past 12-months (*p* < 0.05). Of conflict affected women who experienced past-year physical IPV, 30.54% reported working year-round for cash in the past 12 months, compared to 41.2% of conflict-affected women who did not experience past-year physical (*p* < 0.05). Following a similar pattern, 26.84% of conflict-affected women who reported experiencing past-year sexual IPV and 40.58% of conflict-affected women who did not experience past-year sexual violence reported working year-round for cash in the past 12-months (*p* < 0.05).

In the non-conflict affected group, significant differences were identified between work experiences for women who reported any lifetime IPV, lifetime emotional IPV, any past-year IPV, past-year emotional IPV, and past-year physical IPV. Among non-conflict affected women, 45.73% who experienced any lifetime IPV and 50.58% who did not experience any lifetime IPV reported working year-round for cash in the past 12-months (*p* < 0.01). Similarly, 44.81% of non-conflict affected women who experienced any lifetime emotional IPV and 50.61% of non-conflict affected women who did not experience any lifetime emotional IPV reported working year-round for cash in the past 12-months (*p* < 0.001). When considering past-year IPV among non-conflict affected women, 43.34% of women who reported any past-year IPV and 51% of those who did not experience any past-year IPV reported working year-round for cash in the past 12-months (*p* < 0.001). Non-conflict affected women who experienced past-year emotional IPV were less likely to report working year-round for cash in the past-12 months compared to non-conflict affected women who did not experience past-year emotional IPV, with 43.88% and 49.62%, respectively (*p* < 0.001). Finally, among non-conflict affected women, 42.99% who reported experiencing past-year physical IPV and 49.62% who did not experience any past-year physical IPV indicated having worked year-round for cash in the past 12-months (*p* < 0.01).

### Impacts of IPV on work by conflict-exposure history

Table 4 shows adjusted ORs from multivariable logistic regressions of the impacts of lifetime and past year IPV on engaging in year-round, cash-based employment during the past 12 months for the full sample, and for conflict and non-conflict affected regions. A consistent pattern of increased risk of *not working* (labor force disengagement) was found across all types of and measures of IPV (except sexual IPV, which was statistically insignificant) as shown by odds ratios greater than 1.0 in all cells in the table. Most, although not all results, were statistically significant at conventional levels. For all types of lifetime and past year IPV, differences in estimated ORs between conflict and non-conflict affected regions were not statistically different. Thus, further discussion and costing analysis was limited to the full sample.

**Table 4:** Odds ratios of not being engaged in year-round and cash-based employment during the past 12 months based on IPV experience and conflict exposure

	Full sample (*n* = 8905)	Conflict-affected subsample (*n* = 1780)	Non-conflict affected subsample (*n* = 7125)
	aOR [95% CIs]	aOR [95% CIs]	aOR [95% CIs]
*Lifetime IPV*			
Any	1.08**	1.07	1.08*
	**[1.03–1.14]**	[0.97–1.19]	**[1.02–1.14]**
Emotional	1.09**	1.04	1.09**
	**[1.03–1.14]**	[0.93–1.16]	**[1.03–1.16]**
Physical	1.10**	1.11	1.09**
	**[1.03–1.16]**	[0.97–1.26]	**[1.02–1.16]**
Sexual	1.06	1.13	1.02
	[0.96–1.16]	[0.97–1.32]	[0.91–1.15]
*Past Year IPV*			
Any	1.08**	1.06	1.08**
	**[1.03–1.14]**	[0.95–1.17]	**[1.02–1.15]**
Emotional	1.07**	1.04	1.08*
	**[1.02–1.13]**	[0.94–1.15]	**[1.01–1.14]**
Physical	1.10**	1.13	1.08
	**[1.03–1.18]**	[0.99–1.30]	[1.00–1.17]
Sexual	1.07	1.12	1.05
	[0.97–1.18]	[0.96–1.30]	[0.93–1.20]

Separate multivariable logistic regression models are run for each column (sample group) and row (IPV measure). Covariates in adjusted models include all items in Table 1, as described in the Methods. Bolded results reflect a *p*-value lower than 0.05. *** *p* < 0.001, **p < 0.01, *p < 0.05. Cells related to significance that are blank indicate a *p*-value > 0.05. *aOR* adjusted odds ratio, *IPV* intimate partner violence

Among lifetime IPV, odds of not working were 8–10% greater (*p* < 0.01) among women with emotional, physical, or any IPV. Among past year IPV, odds of emotional, physical, or any IPV were similar, ranging from 8–10% greater (*p* < 0.01). For both lifetime and past year IPV, only sexual IPV was not found to have a statistically significant impact on working, although the odds ratios were consistent with the other IPV measures.

### Labor market costs of IPV

The macroeconomic impacts of lifetime IPV faced by women ages 15–49 in Nigeria are shown in Table 5. The estimated total number of female victims of any lifetime IPV is 18,720,467 across ages 15–49 (derived from Table 2). Combined with an average loss of 4.14% in reduced likelihood of working (marginal effects derived from Table 4), the average annual lost female earnings are an estimated $2,917,000,000 USD in economic damages per year. When this figure is analyzed separately based on differential prevalence of lifetime IPV by conflict and non-conflict affected areas, we find totals of $592,000,000 USD for conflict-affected areas and $2,325,000,000 for non-conflict affected areas. Results are also reported separately for 5-year age intervals between 15–49.

**Table 5:** Labor market costs of any lifetime IPV among women ages 15–49 in Nigeria

*Years of Age*	*Population (total)*	*Lifetime IPV victims (total)*	*IPV costs (total)*	*IPV costs, conflict-affected*	*IPV costs, non-conflict affected*
15–19	11,050,575	4,240,106	$660,611,759	$134,121,283	$526,490,475
20–24	9,174,062	3,520,088	$548,432,404	$111,345,971	$437,086,432
25–39	7,610,302	2,920,073	$454,949,608	$92,366,544	$362,583,063
30–34	6,567,795	2,520,063	$392,627,743	$79,713,593	$312,914,150
35–39	5,629,538	2,160,054	$336,538,066	$68,325,937	$268,212,129
40–44	4,795,533	1,840,046	$286,680,575	$58,203,576	$228,476,999
45–49	3,961,527	1,520,038	$236,823,083	$48,081,215	$188,741,869
Total, ages 15–49	48,789,332	18,720,467	$2,916,663,237	$592,158,119	$2,324,505,118

## Discussion

This study highlights the impacts of IPV on labor market outcomes for Nigerian women, ages 15–49 in 2018. The lifetime and 12-month prevalence of IPV was assessed separately for multiple forms of IPV, and separately for conflict and non-conflict affected sub-groups using data from NDHS. We examined several labor market outcomes, finding a consistent and negative linkage between IPV victimization and engaging in year-round and paid employment. When these data were combined with additional population and wage estimates from the UN Gender Development Index, they provided estimates of the total annual labor market impacts of IPV against women in Nigeria.

Substantial costs of IPV were reported—nearly $3.0 billion USD in a single year (see Table 5). Although this figure is staggering and represents lost potential economic output for the entire population and nation, the figure is highly plausible given the total size of the Nigerian economy. In 2021, the World Bank estimated the total economic output of Nigeria at $440.8 billion dollars. Thus, while IPV is highly prevalent and damaging, our estimates represent total annual losses of slightly less than 1% of GDP. While conservative, this figure reinforces the need for practitioners, policymakers, and funders to address IPV not only as a moral imperative, but to pursue a stronger Nigerian workforce and economy. Specifically, expanding the adoption of the Nigerian Violence Against Persons Prohibition Act (VAPP) will promote systematic protection and resources available for survivors [19–21].

Existing evidence of the impacts of IPV and conflict-exposure on labor force engagement or aggregate economic output are limited, but our analysis contributes to the development of this evidence base. A 2019 study estimated that the direct costs attributed to IPV in the Ghanaian labor market, measured by job absenteeism, exceeded $284 million USD (2017 dollars), or 0.6% of the 2017 GDP of Ghana; when these estimates were expanded to include loss due to reduced productivity while at work (presenteeism) and reduced engagement in household and care work, the total cost of IPV in the Ghanaian labor market rose to nearly $521 million USD, or 1.1% of the 2017 GDP [31]. Similar estimates from Vietnam showed a nearly 1% reduction in GDP at factor cost attributed to IPV [32]. These estimates align with our findings, which indicated losses to the Nigerian economy of nearly 1% attributable to IPV. It is worth restating that these costs, while significant, only represent losses due to reduced labor force engagement. These costs do not include other notable labor costs linked to IPV, including absenteeism or lower paying jobs; nor do these costs include non-labor costs such as healthcare and legal costs. Considering the myriad potential impacts, the total aggregate economic costs due to IPV is likely to be much higher. While the literature base documenting the macroeconomic losses due to IPV is still limited, our findings appear to be consistent and demonstrate the substantial losses—experienced at the individual level but accruing nationally—attributable to this violence.

While IPV costing exercises have yet to be fully explored in humanitarian settings, a few relevant patterns are emerging between conflict, IPV, and labor engagement. Evidence shows that conflict tends to be associated with higher levels of IPV [2]. Interestingly, the patterns related to economic engagement for women in humanitarian settings appear to vary by context. In some settings, economic opportunities are negatively impacted for men and women; yet in other settings, conflict can create new opportunities for women to engage in the labor force. This was seen in conflict-affected Nepal, where women had a 10% greater likelihood of working as the civil war progressed, compared to earlier time points [33]. However, such an increase was not found to be the case in our study, which found a significant negative association between being conflict-affected and women’s labor force participation (42.38% of non-conflict affected women reported working year-round for cash in the past 12-months, compared to only 35.17% of conflict-affected women). Despite finding that conflict affected women in Nigeria experienced significantly higher prevalence of multiple forms of IPV and significantly lower rates of labor force engagement than non-conflict affected women, our costing analysis was not powered to examine employment and IPV experience among conflict-affected subgroup; this reflects a critical gap for research to fill regarding labor force engagement among conflict-affected women. While not extrapolated to include monetized costs, a 2019 analysis of Violence against Women and Girls (VAWG) in South Sudan found that over 5,000 person-days of labor time were lost due to female IPV victimization, nearly 3,500 person-days were lost due to female victimization of non-partner sexual violence (NPSV), and over 3,000 person-days were lost due to male perpetration of both IPV and NPSV, all in a single year [7]. Evidence of the economic costs of IPV in humanitarian settings is just beginning to emerge, but existing evidence demonstrates substantial costs of this violence across communities and economies. There is a need for additional costing estimates to be undertaken to further explore the linkages between IPV and its economic impacts in humanitarian settings. Understanding these costs could prove particularly beneficial within countries experiencing protracted crises, like Nigeria, in their National Action Plan development to address conflict-specific needs.

Our study suggests that IPV prevention efforts are critical not only to reducing prevalence of IPV over time, but also to improving the economic health of national economies. Prevention interventions that aim to shift gender and social norms, particularly those which target young adults, have been shown to contribute to significantly lower rates of IPV perpetration and victimization [34]. The SASA! Intervention, for example, which has been implemented in a number of countries throughout eastern and southern Africa, has demonstrated success in shifting harmful attitudes and norms that sustain IPV. Moreover, a cost-effectiveness study of SASA! in Uganda found favorable results of programmatic costs when considering the IPV cases averted [35]. Similarly, the Male Norms Initiative found that community engagement on gender norms and social expectations was effective in reducing rates of IPV, and that the addition of group education to this intervention demonstrated even more effective shifts in gender norms and reduced IPV prevalence [34]. Another intervention in South Africa that combined economic empowerment objectives with IPV and HIV prevention improved all three outcomes; however, the intervention was only effective when gender equity education was paired with microfinance programming. In other words, micro-loan provision alone was not sufficient to improve economic empowerment, IPV, nor HIV-related outcomes [34]. This finding highlights the importance of gender transformative considerations within economic programming, particularly among women and girls who are vulnerable to IPV. A systematic review on IPV prevalence and prevention across LMICs affirmed this consideration, stating that key IPV prevention efforts should focus on engaging men and boys, gender-norm transformation, and engaging with community stakeholders and influential persons [36]. These promising IPV prevention interventions may be well-suited to the Nigerian context, where gender inequitable norms are dominant [37]. Given our findings, such prevention interventions should be further explored in the Nigerian context to alleviate the numerous negative impacts of IPV, including unnecessary losses to the Nigerian economy.

A few important limitations of the work should be noted. First, our estimates relied on the NDHS data, which are a high quality, but imperfect measure of IPV. While DHS data are known as reputable sources of global health survey data and are one of the leading global surveys that inquire about domestic violence (DV), factors in the construction of the survey and data collection practices may introduce bias into the data. For several reasons, the NDHS may be susceptible to underestimating the true prevalence of domestic violence. First, respondents must be in a completely private setting to be eligible for participation in the domestic violence module. While this is understandable and an ethical best-practice, it also means that respondents who experience spousal control or monitoring (forms of domestic violence) may be prevented from participating, introducing selection bias and potentially skewing prevalence estimates. Despite adhering to rigorous ethical best practices for data collection on the sensitive topic of violence, it is unclear the extent that the 10% of missing data reflect skipping or refusing to answer questions about violence that was experienced. These rates of missingness are similar to other years and countries of DHS data. Secondly, the NDHS only includes “ever-married” women as potential respondents for the domestic violence questions as these questions pertain to have the perpetration of violence by current or previous husbands. This, in turn, introduces two concerns: 1) never-married women are not given the opportunity to identify experiences of abuse they may have had outside of marriage but within an intimate partnership, and 2) men and gender diverse persons are not able to disclose domestic violence victimization. Both restrictions are likely to under-ascertain DV and result in artificially lowered DV prevalence estimates compared to a more inclusive questionnaire.

We believe that under-ascertainment is most likely, and that the true labor market impacts of IPV against women in Nigeria are likely larger. Our classification of labor force participation was based on cash-based and year-round earnings; thus, not all forms of work – paid or unpaid – were considered. Importantly, there is an absence of accounting for the productivity loss of unpaid work, including household or childcare activities, of which the burden of responsibility disproportionately impacts women. Moreover, due to available measures in the NDHS data, we were unable to measure the educational impacts of IPV against girls and young women. To the extent that IPV has negative educational impacts at earlier ages, it is likely that we under-ascertain the true costs of IPV by failing to fully control for reduced educational attainment and lifetime earnings. Additionally, respondent-specific earnings were not available in the NDHS data, so our costing estimates relay on median earnings from the UN Gender Development Index. While this is appropriate for aggregate costing, it adds an element of measurement error due to the design of the NDHS. Moreover, the cross-sectional nature of the NDHS data does not permit true causal inference or more robust statistical methods. Thus, these results are best considered to be an estimate of the association between IPV and negative labor market impacts. Future studies using alternative data sources may be needed to more rigorously advance statistical estimates of the labor market impacts of IPV.

## Conclusions

Using multiple data sources, this work has quantified the impacts of IPV on employment and labor market engagement for working age Nigerian women in 2018. Despite using conservative assumptions, estimated annual costs approach $3.0 billion USD, about 1% of Nigeria’s total economic output. This is a societal cost, not strictly limited to women, but borne by families, government, and all of Nigeria. If stronger funding and prevention measures could reduce the incidence of IPV against women in Nigeria, a substantial portion of these costs could be reclaimed, resulting in a stronger Nigeria workforce and economy for the twenty-first century.

Because labor market costs are large, there is almost surely a positive return on investment and economic case to be made for stronger protections for girls and women in Nigeria. Specific policy evaluations, cost-effectiveness, and cost–benefit analyses may be important additional steps in the future for identifying the most efficient strategies for reducing or eliminating these costs. Although labor market impacts are costly, we reiterate that health costs and other social costs were not included in this analysis and would make the benefits of prevention appear even larger. Finally, we remind readers that the human and moral imperative for protecting the quality of life and well-being of women remains salient and fundamental alongside the economic case.

## Data Availability

The data that support the findings of this study are publicly available.
